# Characterization of lower-extremity biomechanics when walking with a hands-free crutch: an integrated experimental and computational analysis

**DOI:** 10.3389/fbioe.2026.1941015

**Published:** 2026-09-30

**Authors:** Md. Arifuzzaman Arif, Jaques Reifman, Ankur Padhye, Stacey Meardon, John Willson, Jose E. Rubio

**Affiliations:** 1 Department of Defense Biotechnology High Performance Computing Software Applications Institute, Defense Health Agency Research & Development, Medical Research and Development Command, Fort Detrick, MD, United States; 2 The Henry M. Jackson Foundation for the Advancement of Military Medicine, Inc., Bethesda, MD, United States; 3 Department of Physical Therapy, East Carolina University, Greenville, NC, United States

**Keywords:** computational models, hands-free crutch, leg stabilization, mobility-aid device, weight-bearing

## Abstract

**Background:**

Lower-leg wounds and fractures are some of the most common types of traumatic injuries sustained by U.S. Service members during combat operations. Existing guidelines for on-field management of lower-leg injuries recommend stabilizing the injury and preventing weight-bearing on the affected leg. However, adhering to these recommendations could be challenging because the casualty may need to walk independently to obtain medical care. In this exploratory experimental and computational study, we assessed the biomechanical characteristics of a hands-free crutch device to aid in the mobility of healthy individuals with a hypothetical lower-leg injury.

**Methods:**

We conducted an experimental study where we collected anthropometric, body-motion, ground reaction force, spatiotemporal, and electromyography data from 10 young, healthy men who walked with and without the Freedom Leg hands-free crutch on an instrumented treadmill. Using these data, we developed individualized musculoskeletal and finite-element models and computed biomechanical parameters, including joint kinematics and kinetics, muscle activity and metabolic power demand, and tibial stress, for each participant under each of the two walking conditions. We evaluated the suitability of the device by comparing the model-predicted biomechanical parameters between the two conditions.

**Results:**

Compared to walking unassisted, walking with the Freedom Leg reduced the peak knee joint reaction force (JRF) by 47%, peak ankle JRF by 98%, and peak tibial stress by 59% in the crutch-supported leg. However, it also introduced unintended consequences in the contralateral leg, increasing the peak hip JRF by 19%, peak hip flexion moment by 50%, and peak hip extension moment by 89% as well as the mean metabolic power demand in representative hip and knee muscles.

**Conclusion:**

The Freedom Leg improved mobility by effectively reducing weight-bearing and stabilizing the crutch-supported leg. However, changes in gait patterns also introduced unintended biomechanical consequences in the contralateral leg. This exploratory study provides insights into the biomechanical effects of walking with the Freedom Leg in healthy, young individuals and may inform the development and assessment of future hands-free crutch devices to improve mobility of Service members with lower-leg injuries.

## 1 Introduction

Extremity wounds and fractures constitute the majority of traumatic injuries in U.S. combat operations (Owens et al., 2007). An epidemiological study of Warfighters deployed to Afghanistan and Iraq between 2001 and 2005 reported 3,575 extremity wounds, of which 13% (454) were lower-extremity injuries, including 218 fractures to the tibia and fibula (Owens et al., 2007). Current guidelines for on-field management of leg fractures recommend stabilizing the fracture and avoiding weight-bearing on the injured leg (Joint Trauma System, 2022). However, during combat, it may not always be possible to adhere to these recommendations, especially if the casualty collection point or Role 1 site are far from the point of injury. In addition, Service members who sustain a lower-leg injury on the battlefield are typically carried on a litter by four to six military personnel, making the transport of lower-extremity fractures particularly resource intensive (Johnson et al., 2023). Ideally, solutions would not only provide independent mobility to Service members with a lower-limb wound or fracture but also minimize further injury while preserving Force Readiness.

From a clinical perspective, lower-extremity injuries accounted for approximately 15% (119,815 patients) of U.S. emergency department visits in 2009, including injuries to the ankle (20%), knee (16%), foot (15%), lower leg (11%), and toe (7%) (Lambers et al., 2012). Patients with these injuries often require a period of mechanical unloading and rehabilitation. For centuries, axillary crutches have served as the standard assistive and mobility-aid device for non-weight-bearing or partial weight-bearing during the rehabilitation period after a lower-extremity injury, with more than 575,000 standard axillary crutches prescribed annually in the U.S. (Reynders-Frederix et al., 2020). Although these crutches are easy to use and economical, they are not suitable for all patients. For example, standard crutches are not practical for patients with a concomitant upper-extremity injury, as approximately 44% of the patient’s body weight is transferred to the arm during crutch stance (Goh et al., 1986), causing additional strain on the upper extremities and possibly leading to pain, weakness, or numbness in the shoulder and arm (i.e., brachial plexus neuropathy) after prolonged use (Raikin and Froimson, 1997). In addition, Rasouli et al. reported that proper adherence to axillary crutch gait is challenging for inexperienced users (Rasouli and Reed, 2020).

In addition to the standard axillary crutches, there are various other types of mobility-aid devices, including hands-free crutches, which have improved performance characteristics (Rambani et al., 2007; Martin et al., 2019; Dewar and Martin, 2020; Orishimo et al., 2021; Saffuri et al., 2024). For example, compared to a standard crutch, the use of a hands-free crutch results in higher functional outcome scores during rehabilitation (Rambani et al., 2007), lower hip joint reaction forces (JRFs) (Orishimo et al., 2021), lower heart rate and metabolic demand (Martin et al., 2019; Hegeman and Huh, 2024), lower rating of perceived exertion (RPE) (Martin et al., 2019; Canter et al., 2024), lower limb angular velocity (Wiederien et al., 2024), and lower whole-body angular momentum (Wiederien et al., 2024). Hands-free crutch devices work by primarily offloading the distal part of the lower leg, while transmitting load through the proximal shank and, as such, are particularly useful for patients with ankle or foot injuries (Rambani et al., 2007; Martin et al., 2019; Orishimo et al., 2021). Furthermore, in an austere operational environment, a hands-free crutch could allow for independent locomotion of Service members who sustain a tibial fracture or other lower-leg injury, provide enhanced support during rehabilitation by offloading and stabilizing the injured leg, and accelerate return to duty.

Previous studies have assessed changes in biomechanical gait parameters when healthy participants walked with and without different types of hands-free devices (Dewar and Martin, 2020; Dewar et al., 2021; Orishimo et al., 2021; Kim et al., 2023; Saffuri et al., 2024; Wiederien et al., 2024). By and large, these studies assessed spatiotemporal parameters, plantar force, ground reaction force (GRF), hip and lower-back JRFs, lower-limb joint angles, shank angular velocity, and electromyography (EMG) measures (Dewar and Martin, 2020; Dewar et al., 2021; Orishimo et al., 2021; Kim et al., 2023; Saffuri et al., 2024; Wiederien et al., 2024). However, it is unclear how GRF is transmitted through the lower-extremity joints and the shank, and whether hands-free crutches are as beneficial for wounds or fractures of the tibia, because these devices have been reported to cause discomfort in the shank region of the leg (Saffuri et al., 2024). In addition, some of these studies only assessed the effect on the injured leg (Dewar and Martin, 2020; Dewar et al., 2021), while it has been reported that their use also affects the biomechanics on the contralateral limb due to compensatory effects (Saffuri et al., 2024). Moreover, the majority of these studies have been limited to the iWalk device (Rasouli and Reed, 2020; Hegeman and Huh, 2024), and none performed a comprehensive analysis of the kinetics (i.e., moments and JRFs) of individual joints of the lower leg, limiting our understanding of the applicability of hands-free crutches for knee and shank injuries.

To address this research gap, we conducted a comprehensive biomechanical investigation using integrated musculoskeletal (MSK) and finite-element (FE) models to characterize the biomechanics of all three lower-extremity joints (hip, knee, and ankle) in both the crutch-supported and the contralateral limb due to walking with a hands-free crutch. The goal of this exploratory interdisciplinary study was to evaluate the suitability of a commercial off-the-shelf hands-free crutch device to aid mobility after an injury to the lower-leg complex. We conducted an experimental study to collect anthropometric, body-motion, GRF, spatiotemporal, and EMG measurements from 10 healthy participants walking with and without the device on an instrumented treadmill and used these data to develop integrated individualized MSK and FE models to compute joint kinematics, joint kinetics, muscle activity and metabolic power demand, and tibial stress for each participant under each of the two walking conditions. Using the model predictions, we evaluated the suitability of the device for its capability to avoid weight-bearing and stabilize the tibia on the crutch-supported leg as well as to identify unintended consequences on the contralateral leg. We hypothesized that the use of a hands-free crutch device would reduce the JRFs and moments at the knee and ankle joints as well as decrease the stress in the tibia of the crutch-supported leg. In addition, we hypothesized that the device would increase the JRFs, moments, and muscle metabolic power demand in the contralateral leg.

## 2 Methods

In this interdisciplinary work, we first conducted an experimental study to collect data from participants walking with and without a hands-free crutch on an instrumented treadmill (Figure 1A). Using these data, we then developed individualized MSK and FE models to compute joint kinematics, joint kinetics, muscle activity and metabolic power demand, and tibial stress for each participant under each of the two walking conditions (Figures 1B,C).

**FIGURE 1:**
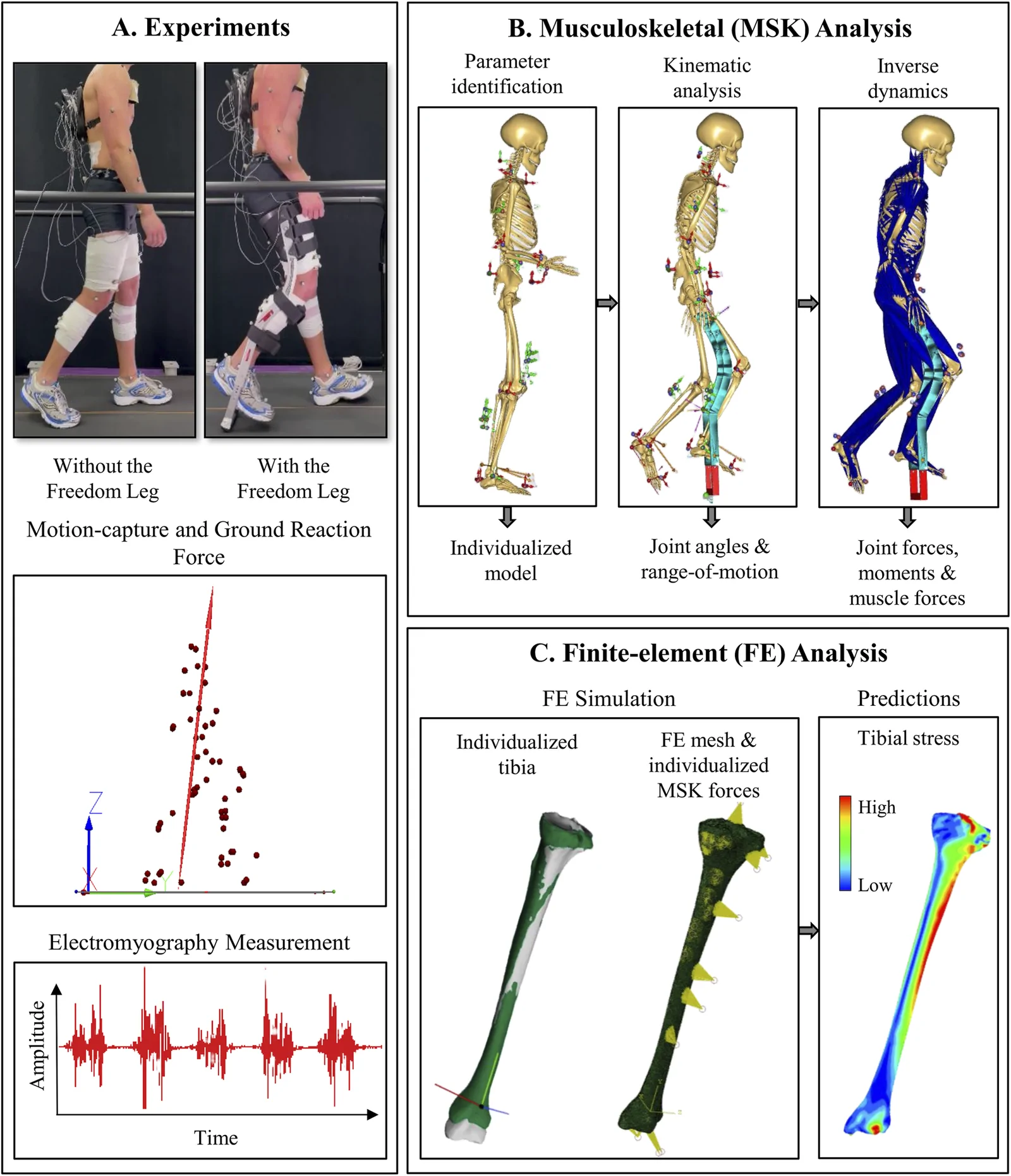
Summary of the integrated interdisciplinary experimental and computational study design. **(A)** We performed a laboratory study and collected motion-capture, ground reaction force, and electromyography data from 10 participants as they walked on an instrumented treadmill with and without the Freedom Leg hands-free crutch device. **(B)** We developed individualized musculoskeletal (MSK) models by incorporating participant-specific anthropometric measurements and used them to conduct simulations to compute joint angles and range of motion, joint reaction forces and moments, and muscle forces and metabolic power demand for each participant under each of the two walking conditions. **(C)** For each participant and walking condition, we developed finite-element models of the right tibia using the participant-specific scaled geometry from the MSK analyses and predicted the stress distribution throughout the tibia.

### 2.1 Experimental study

#### 2.1.1 Hands-free crutch selection

We conducted a preliminary investigation to select a hands-free crutch device for our study. Briefly, we identified two commercially available hands-free crutches, the XLeg (Robinson Exotech Corp., Alberta, Canada) (Robinson Exotech Corporation, 2026) and the Freedom Leg 3.0 (Forward Mobility, Jacksonville, FL, United States of America) (Forward Mobility Inc., 2026), and compared them based on fitting-time and gait-performance metrics, including cadence and gait speed. To this end, we recruited a healthy participant (32-year-old male; height: 1.66 m; mass: 60 kg) and conducted three 20-s treadmill-walking trials with each device worn on the right leg (walking speeds of 0.45, 0.50, and 0.60 m/s). Compared with the XLeg, walking with the Freedom Leg device resulted in shorter fitting time (7 vs. 15 min), faster speed (0.60 vs. 0.50 m/s) as the participant failed to reach 0.60 m/s with the XLeg device, and higher cadence (77 vs. 70 steps/minute). Based on these results, we selected the Freedom Leg device for the study.

#### 2.1.2 Participants and data collection

We enrolled 10 young, healthy men (18–30 years old) demographically representative of male recruits in accordance with an anthropometric survey of U.S. Army Soldiers (Silsbury et al., 2015). We included participants who had no self-reported history of health conditions or impairments restricting their ability to exercise and who engaged in vigorous-to-moderate physical activity at least three times per week for 1.0–1.5 h each session. We excluded participants who had any injury that limited their ability to be physically active in the 3 months prior to study participation or who were not comfortable walking on a treadmill. We also excluded participants whose height (<1.32 m or >1.93 m) or inseam (>0.86 m) exceeded the hands-free crutch manufacturer’s recommendations. The Institutional Review Board at East Carolina University and the Office of Human Research Oversight at Fort Detrick, MD, approved the study protocol, and we performed all methods in accordance with the relevant guidelines and regulations. After participants signed an informed consent form, we recorded their age, height, mass, and body fat percentage (Table 1).

**TABLE 1:** Anthropometric measurements of the 10 young, healthy men in our study.

​	Age (years)	Height (m)	Mass (kg)	Body fat (%)	BMI (kg/m^2^)
Mean (±SD)	21.1 (1.4)	1.76 (0.06)	75.3 (9.1)	13.1 (3.0)	24.3 (2.9)
Range	19–24	1.66–1.88	54.4–87.3	8.7–19.7	17.3–27.5

The data are presented as means [±1 standard deviation (SD)] or a range. BMI: body mass index.

Each participant completed two walking trials, one with and another without the device, in the same order during a single visit. Each trial involved walking at a constant preferred speed for 40 s on a flat-level, dual-belt instrumented treadmill (Bertec FIT, Bertec Corp., Columbus, OH, United States of America). Each 40-s trial consisted of two 20-s segments with about 2 min in between them, which we used to verify the functionality of the data-collection system. Participants completed the trials in the following sequence: *1*) walking with the hands-free crutch (Freedom Leg 3.0) and *2*) walking without the crutch. Participants rested for at least 5 min between trials but were allowed additional time as needed before starting the second trial. The rationale for completing the walking trials in this order was to first determine the preferred walking speed with the hands-free crutch for each participant and then to use the same speed during the subsequent walking trial without the device. This approach allowed us to minimize potential confounding effects of walking speed on gait biomechanics and attribute the observed biomechanical differences primarily to the use of the hands-free crutch.

Before the first trial, we fitted and adjusted the hands-free crutch to each participant according to the manufacturer’s recommendations. Briefly, with the participant seated, we secured the crutch to the right leg using the upper-leg (thigh) and lower-leg (calf) straps and adjusted the footrest position to maintain approximately 5 cm between the ground and the base of the foot with the ankle in a neutral position. Then, we asked the participant to stand while we observed their bilateral anterior iliac spine for signs of pelvic asymmetry, making any necessary adjustments to the upper-leg strap on the crutch to ensure a level pelvis. Subsequently, to acclimatize to the hands-free crutch, participants walked with the device on a 25-m level walkway, with assistance from a researcher as needed, until they could walk independently and comfortably. Participants repeated the same acclimation process on a treadmill, starting at a speed similar to that observed during overground walking. We incrementally increased the treadmill speed until participants reached a comfortable cadence, which we recorded as their preferred walking speed for the two trials. After acclimation, participants completed the walking trial with the Freedom Leg device, followed by a trial without the crutch at the matched preferred walking speed.

During each walking trial, we synchronously collected motion-capture data, GRFs, and EMG data (Figure 1A) for 40 s. We placed reflective spherical markers at 49 anatomical landmarks (head, torso, back, arms, and legs) and collected motion-capture data at a sampling frequency of 200 Hz, using a 17-camera system (Qualisys AB, Göteborg, Sweden). For trials with the hands-free crutch, we also placed seven reflective markers on the side rails, footrest, and upper- and lower-leg straps of the crutch to track their location (Figure 2A). We measured GRFs from the instrumented treadmill at a sampling frequency of 2,000 Hz. In addition, we collected bilateral EMG data with the same frequency to record muscle activity from five muscle groups [iliocostalis (erector spinae), gluteus medius, biceps femoris, rectus femoris, and medial gastrocnemius] using pre-gelled snap Ag/AgCl electrodes (Kendall 530 Series Foam Electrodes, Cardinal Health, Dublin, OH, United States of America) placed bilaterally on muscle-specific locations in accordance with the Surface EMG for Non-Invasive Assessment of Muscles (SENIAM) program (Hermens et al., 2000). We collected these data using a total system gain of 4,000× and a band-pass filter (20–500 Hz) (Motion Lab Systems, Lake Elsinore, CA, United States of America). As part of each of the two walking trials, we measured marker location with participants standing still to collect calibration data (i.e., to perform a static trial) for each participant.

**FIGURE 2:**
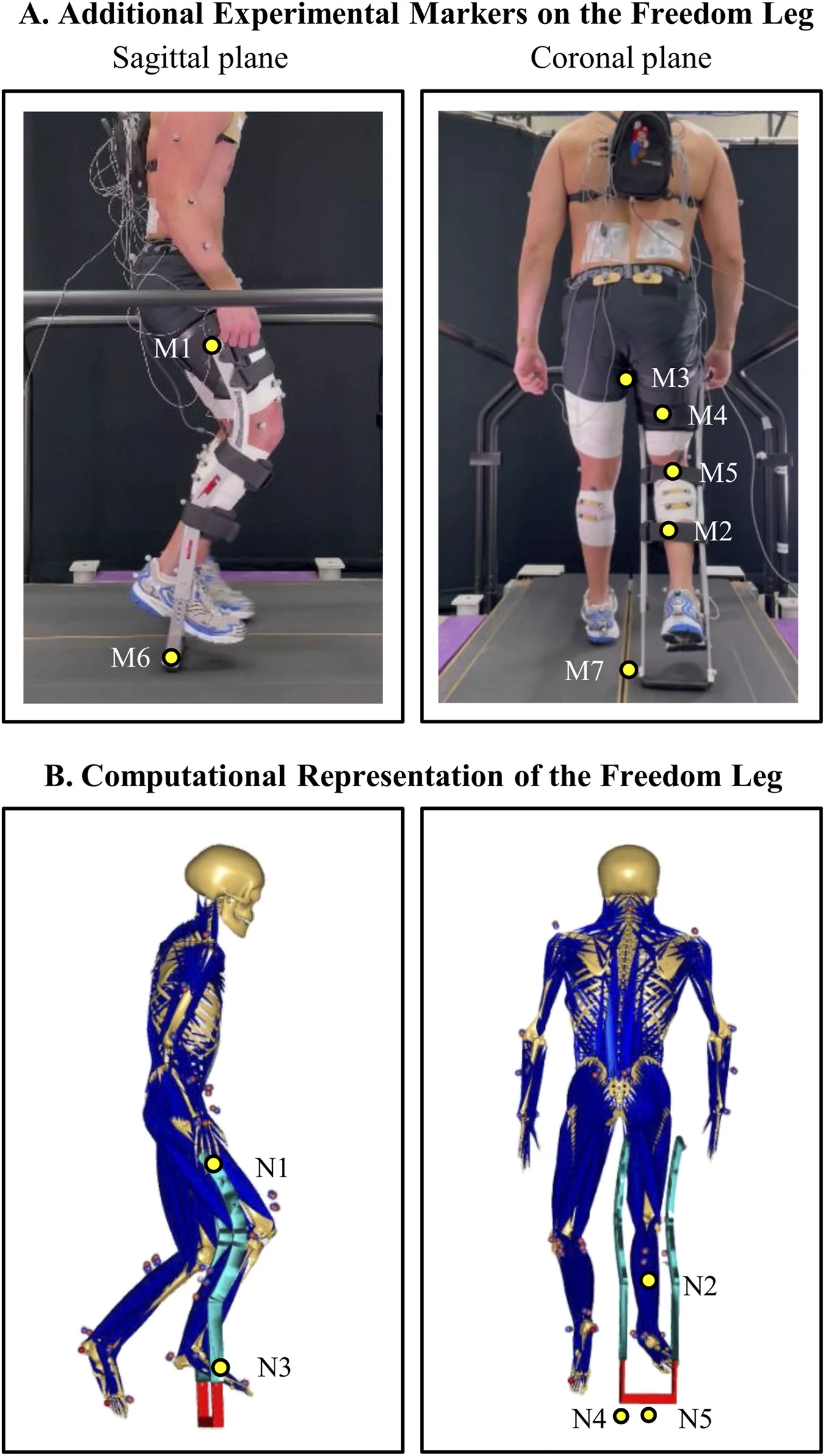
**(A)** Experimental setup describing the locations of the seven reflective markers placed on the Freedom Leg for motion capture during trials in which participants wore the hands-free crutch device. We affixed two markers to the crutch side rails [one on the lateral side (M1) and one on the medial side (M3)], one marker to the posterior side of the upper-leg strap (M4), two markers to the posterior side of the lower-leg straps (M2 and M5), and two markers to the footrest [one on the lateral side (M6) and one on the medial side (M7)]. **(B)** Representation of the Freedom Leg in the musculoskeletal (MSK) model using two rigid-body segments to characterize the crutch side rails and the footrest. We defined two nodes (N1 and N2) to couple the side rails to the upper and lower leg, respectively, in the MSK model. In addition, we rigidly connected the side rails to the footrest at a participant-specific location (node N3). Finally, we defined two nodes (N4 and N5) on the footrest to represent its interaction with the ground.

We used Visual3D software (HAS-Motion, Kingston, Ontario, Canada) in combination with a custom LabVIEW program (National Instruments, Austin, TX, United States of America) to remove any direct-current offset caused by electrical drift from the GRF data and to apply a zero-lag, fourth-order Butterworth filter with a 10-Hz cutoff frequency to the marker data. For each walking trial, we used the GRF and the treadmill-speed data to calculate the step length and step time.

### 2.2 Computational analyses

#### 2.2.1 Individualized musculoskeletal models

In our previous work (Xu et al., 2016; Rubio et al., 2023; Tong et al., 2023; Nagaraja et al., 2025; Rubio et al., 2026), using the AnyBody Modeling System (AnyBody Technology, Aalborg, Denmark), we developed and validated participant-specific MSK models to predict joint kinematics, joint kinetics, and muscle activity under each walking condition (Figure 1B). Briefly, we started with a generic MSK model available in the AnyBody software consisting of rigid segments to represent the arms, legs, feet, trunk, and pelvis as well as 169 muscles per leg in the lower extremities and 188 muscles in the trunk (Damsgaard et al., 2006). Next, we individualized the MSK model to each participant by scaling the generic model using their anthropometric measurements. We defined 49 markers in the MSK model to match the marker configuration used in the experimental trials. Then, we optimized the length of each body segment by minimizing the distance between the experimental markers and the modeled marker positions using the data from the static trial (Damsgaard et al., 2006; Lund et al., 2015).

For trials that involved participants wearing the Freedom Leg device, we modeled the hands-free crutch using rigid body segments in the AnyBody software. Specifically, we modeled the Freedom Leg as two rigid body segments (Figure 2B), with a total weight of 3.0 kg that accounted for the straps’ weight, using the mass and inertia tensor derived from the corresponding computer-aided design model. The first segment represented the side rails of the crutch. Because we assumed no relative motion between the side rails, we modeled them as a single rigid body segment. We coupled this segment to the right leg in the MSK model using node N1 on the upper leg and node N2 on the lower leg, corresponding to the locations of experimental markers M1 and M2, respectively (Figure 2). In addition, based on our observations during the trials, we constrained the side rails and the upper leg for relative translation and allowed them to rotate relative to each other. Similarly, between the side rails and the lower leg, we allowed relative translation only along the longitudinal axis of the lower leg, while constraining rotation only about that same axis. The second segment represented the footrest, which we rigidly attached to the side rails at a participant-specific location recorded during the experiments to maintain an ∼5.0-cm clearance between the ground and the base of the foot (node N3, Figure 2B). In addition, we defined two contact nodes on the footrest (nodes N4 and N5) to replicate the experimentally observed contact pattern between the footrest and the ground. For the crutch-assisted right leg, we applied the GRF to the Freedom Leg using these two contact nodes on the footrest, and for the contralateral leg, we applied the GRF directly to the left leg to represent the actual walking condition.

To compute the joint kinematics [i.e., angles and range of motion (ROM)] at the hip, knee, and ankle, we used the motion-capture data collected for each walking trial as input to the participant-specific MSK models. Then, using the AnyBody software, we conducted inverse dynamic analyses to compute the joint kinetics (forces and moments) at these same locations as well as the muscle forces in the leg and back muscles. To perform these analyses, the software established a coordinate system at the proximal end of each body segment and applied the predicted joint kinematics and the measured GRFs to the Newton–Euler equations to compute JRFs, joint moments, and muscle forces. To calculate the muscle forces, the software used an optimization-based recruitment algorithm to identify the muscle activation pattern required to balance the external joint loads at each timepoint (Rasmussen et al., 2001; Damsgaard et al., 2006). This optimization algorithm determined the muscle force distribution according to the minimum/maximum criterion, which minimized the maximum muscle activity across all muscles subject to the inverse dynamics equilibrium equations that reproduced the net joint moments computed from the measured motion (Rasmussen et al., 2001). Within this optimization procedure and for each timepoint, the activity of a given muscle was defined as the force produced by the muscle normalized by its maximum strength. In addition, we computed the metabolic power demand of representative major hip and knee muscle groups based on the metabolic energy model in the AnyBody software, using the predicted muscle forces to compute instantaneous metabolic power demand for each muscle.

We conducted stride analyses using the outputs of the MSK models. Based on our previous work (Xu et al., 2016; Rubio et al., 2023; Tong et al., 2023; Nagaraja et al., 2025), we identified a stride by determining the prominent peaks in the vertical GRF and defining stride boundaries (start and end frames) using the zero-force intervals immediately preceding and following each peak. This approach yielded approximately 20–30 strides for each 40-s walking trial. Then, we resampled each output to a uniform length consisting of 100 evenly spaced values, representing 0–100% of the stride cycle. Finally, to reduce inter-participant variability associated with differences in body height and weight, we normalized the step length by body height, the GRFs and JRFs by body weight, and the joint moments and metabolic power demand by body mass (Wannop et al., 2012).

#### 2.2.2 Individualized finite-element models of the tibia

We developed a participant-specific FE model to predict the peak stress in the right tibia. For this model, we focused our analyses exclusively on the tibia of the right leg because it was the leg supported by the crutch. During the parameter identification procedure in AnyBody, we optimized the dimensions of the tibia bone to match the participant’s anthropometric measurements and motion-capture data (Lund et al., 2015). To develop the FE model, we first exported the optimized (i.e., the scaled) geometry of the tibia from the participant-specific MSK model to the HyperMesh software (Altair Engineering, Inc., Troy, MI, United States of America) to generate a three-dimensional (3-D) mesh consisting of 10-noded quadratic tetrahedral elements with an average element size of 2.0 mm. To determine the element size, we previously performed a mesh-convergence analysis by systematically decreasing the element size of the tibia and evaluating the tibial cumulative stress resulting from running with no load at a preferred speed (Xu et al., 2017). From the mesh-convergence analysis, we determined that the peak tibial cumulative stress increased marginally with further mesh refinement below an element size of 3.5 mm. Therefore, to conservatively meet this element-size criterion, we selected a 2.0-mm element size for the present study to maintain solution accuracy, while achieving an acceptable computational cost. In addition, we used the HyperMesh software to assess mesh quality and generate tetrahedral elements with a maximum aspect ratio of 5.0, minimum tetrahedral collapse value of 0.3, and maximum volumetric skewness of 0.9.

We divided the 3-D mesh into trabecular and cortical bone elements by assuming a uniform cortical thickness of 4.0 mm (Maeda et al., 2020; Taylor et al., 2021). For the trabecular bone elements, we assigned a Young’s modulus of 0.1 GPa, a density of 1,006 kg/m^3^, and a Poisson’s ratio of 0.2. For the cortical bone elements, we assigned a Young’s modulus of 12.0 GPa, a density of 1,660 kg/m^3^, and a Poisson’s ratio of 0.3 (Wong et al., 2010; Rubio et al., 2025). We performed FE simulations to predict the peak stress in the right tibia for each participant under each walking condition. To this end, we applied the muscle forces and joint forces computed from the MSK simulations as participant-specific loading conditions (i.e., the inputs) to the tibial FE model. These are two distinct types of loads, where the joint forces represent the net force transmitted across the articulating joint without accounting for internal muscle forces, while the muscle forces represent the loads applied through the corresponding muscle insertion points on the tibial surface. Together, these loads define the pathways through which forces are transmitted to the tibia. We did not fix the proximal and distal ends of the tibia in the FE model. The force and moment outputs from the MSK analyses guided the FE simulations, where we mapped each joint force and moment into the tibia using a coupling constraint between a reference node and the tibial surface. We distributed the forces (and moments) over a small area rather than concentrating them at a single node. Using the ABAQUS 2023 software (Dassault Systèmes, Vélizy-Villacoublay, France), we performed FE analyses and computed the von Mises stress (90th percentile) on each element of the tibia.

#### 2.2.3 Statistical analysis

We performed a power analysis to determine the sample size needed for our study. We based our analysis on the study by Dewar et al. (2021), who measured changes in lower-leg muscle activity while subjects walked along a 20-m walkway with a hands-free crutch. Using the test for equality proposed by Chow et al. (2002), an effect size of 1.4 (Dewar et al., 2021), and considering the null hypothesis that walking with a hands-free crutch did not change the lower-leg muscle activity compared to walking without the device, we estimated a minimum sample size of eight participants to achieve 80% power at a significance level of 5%. We used a conservative sample size of 10 for this study.

Overall, we analyzed a total of 35 parameters for the left leg and 36 parameters for the hypothetically “injured” right leg. These included two spatiotemporal parameters estimated from the experiment and 26 biomechanical parameters computed by the personalized MSK models to characterize the spatiotemporal, kinematic, and kinetic changes of each participant’s gait (both legs) while walking with and without the hands-free crutch device. In addition, we analyzed the metabolic power demand of seven representative major hip and knee muscle groups for each leg. For the right leg, using the FE model, we also evaluated the peak von Mises stress in the tibia. For each participant under each of the two conditions (walking with and without the device), we calculated the mean and standard deviation (SD) of each biomechanical and physiological parameter across the ∼20–30 strides. Because some parameters were not normally distributed based on Shapiro-Wilk’s test for normality (Shapiro and Wilk, 1965), we used the non-parametric Wilcoxon signed-rank test (Wilcoxon, 1992) with Benjamini−Hochberg correction for multiple comparisons (Benjamini and Hochberg, 1995) to test for statistically significant differences in each parameter between the two conditions. We considered differences with *p*-values smaller than 0.05 as statistically significant. Similarly, we computed the effect size using the non-parametric rank-biserial correlation *r* as a measure of probabilistic dominance of one condition over the other (Kerby, 2014), where we interpreted the extent of the correlation *r* according to the guidelines suggested by Peres (Fiel Peres, 2026) and adapted from Vargha and Delaney (2000): 0.11 ≤ |*r*| < 0.28 (small), 0.28 ≤ |*r*| < 0.43 (medium), and 0.43 ≤ |*r*| ≤ 1.00 (large).

Using a selected subset of the original list of 36 biomechanical and physiological parameters, we evaluated the performance of the Freedom Leg for its ability to achieve two main objectives: *1*) avoid weight-bearing (right leg) and *2*) stabilize the tibia (right leg). In addition, we evaluated potential unintended consequences on the left leg. We assessed the amount of weight-bearing in the right leg by comparing the stress in the tibia and the JRFs at the ankle and knee when participants walked with and without the Freedom Leg. We determined that the Freedom Leg avoided weight-bearing if its use reduced the group mean value of each of these three parameters by at least two standard error of the mean (SEM) of the corresponding mean in the unassisted condition. As such, we used the SEM to characterize the uncertainty around the computed group mean for each of the three biomechanical parameters under the two conditions and to facilitate group-level comparisons across the two conditions. The JRFs represent the net force acting across a joint, integrating both the external load (i.e., the GRF transmitted upward through the lower limb during the stance phase) and the internal muscle forces (Winter 1984; Nigg and Herzog, 1999). We evaluated the ability of the Freedom Leg to stabilize the right tibia by comparing the model-predicted moments at the knee and ankle when participants walked with and without the device as well as the peak stress in the tibia (Clark et al., 2004; Saadat et al., 2026).

We determined that the Freedom Leg stabilized the tibia if its use reduced the group mean value of each of these parameters by at least two SEM of their values when participants walked without the device. Joint moments reflect the net mechanical demand required to counteract external loads (i.e., the GRFs) and maintain controlled and stable movement. Increases in the magnitude or variability of these moments may reflect elevated muscular demands for joint stabilization, whereas consistent moment patterns across consecutive strides indicate more stable and efficient motor control strategies (Sloot and van der Krogt, 2018). Von Mises stress is a scalar measure of multiaxial stress state, reflecting the combined effect of normal and shear stresses in terms of an equivalent stress value (Kozin et al., 2026). Therefore, a lower von Mises stress in the tibia indicates a reduced overall stress state, suggesting a lower mechanical demand on the bone. Reducing the overall stress state limits excessive strain on the fractured bone, thereby stabilizing the fracture site, enhancing mechanical stability, and creating a biomechanical environment conducive to fracture healing (Hast et al., 2024). Finally, we assessed whether there were any unintended consequences in the contralateral leg resulting from walking with the Freedom Leg by identifying biomechanical parameters that changed significantly (*p*-value <0.05 after correction for multiple comparisons) and exhibited a rank-biserial correlation *r*, with 0.80 ≤ |*r*| ≤ 1.00.

## 3 Results

### 3.1 Muscle activity and model assessment

To assess the individualized MSK models for participants walking on a treadmill with and without the Freedom Leg device, we compared the time courses of the model-predicted muscle activity with the measured EMG recordings for three representative muscles: the iliocostalis (erector spinae) in the lower back, the gluteus medius in the upper leg, and the medial gastrocnemius in the lower leg. We selected these three bilateral muscles for model assessment because they represent the major anatomical regions and functional roles affected by crutch-assisted walking. Specifically, the iliocostalis reflects the trunk stabilization and potential compensatory loading in the lower back, the gluteus medius captures the compensatory loading in the contralateral limb, and the medial gastrocnemius represents the lower-limb function and offloading effects of the crutch-assisted gait. Additionally, we selected a representative participant whose height was closest to the group mean. Although both the model-predicted muscle activity and the EMG recordings can serve as proxies for relative muscle recruitment, our analysis is limited to qualitative trend assessments because MSK models and EMG recordings yield different parameters, which cannot be directly compared. While the MSK model predicted muscle activity as the force produced by the muscle normalized by its maximum strength, EMG measured the electrical signal generated by the muscle fibers in response to neural excitation. In addition, other factors further preclude a direct quantitative comparison, including electromechanical delays of EMG sensors (Corcos et al., 1992), differences in the relationship between neural activation magnitude and muscle forces, assumed to have a more direct association in MSK models dictated by the force-length-velocity relationship (Zajac, 1989) but a nonlinear association in EMG recordings (Roberts and Gabaldón, 2008), and potential noise and sensor-signal artifacts influenced by external factors that MSK models do not account for, including crosstalk from adjacent muscles, electrode detachment, and skin impedance variations (Corcos et al., 1992; Damsgaard et al., 2006; Roberts and Gabaldón, 2008).

Figure 3 shows the EMG recordings (solid line) and the model-predicted muscle activities (dashed line) for both legs of a representative participant walking with and without the Freedom Leg device. The plots show the mean ± one SD for the 20–30 strides recorded over the 40-s walking trial using two distinct y-axis scales to illustrate qualitative trends. Overall, we observed reasonable agreement in the trends of the predicted and measured values for each of the three muscles in both legs and the lower back. For walking without the device (Figure 3A), we observed that the predicted muscle activity was generally consistent with the EMG recordings in terms of onset of muscle activation during the stance phase (0–∼70% of the stride) and the swing phase (∼70–100% of the stride). For example, the activation and deactivation trends of the iliocostalis muscle matched almost perfectly when we ignored the offset in magnitude between the two quantities. For walking with the device (Figure 3B), consistent with the EMG recordings, the model predictions yielded increased muscle activity in the gluteus medius and medial gastrocnemius muscles in the left leg compared to the right leg (i.e., the crutch-supported leg). The increased gluteus medius muscle activity in the left leg was likely the result of contralateral hip loading, which was consistent with the contralateral hip discomfort experienced by the participants when they wore the device. In addition, the model predictions exhibited negligible muscle activity in the medial gastrocnemius muscle of the right leg, suggesting that the use of the device resulted in substantial weight-offloading in the lower leg, which was also in agreement with the EMG recordings. Overall, we observed similar trends in the corresponding comparisons between experimentally measured EMG recordings and model-predicted muscle activities for the remaining nine participants (Supplementary Figures S1-S9).

**FIGURE 3:**
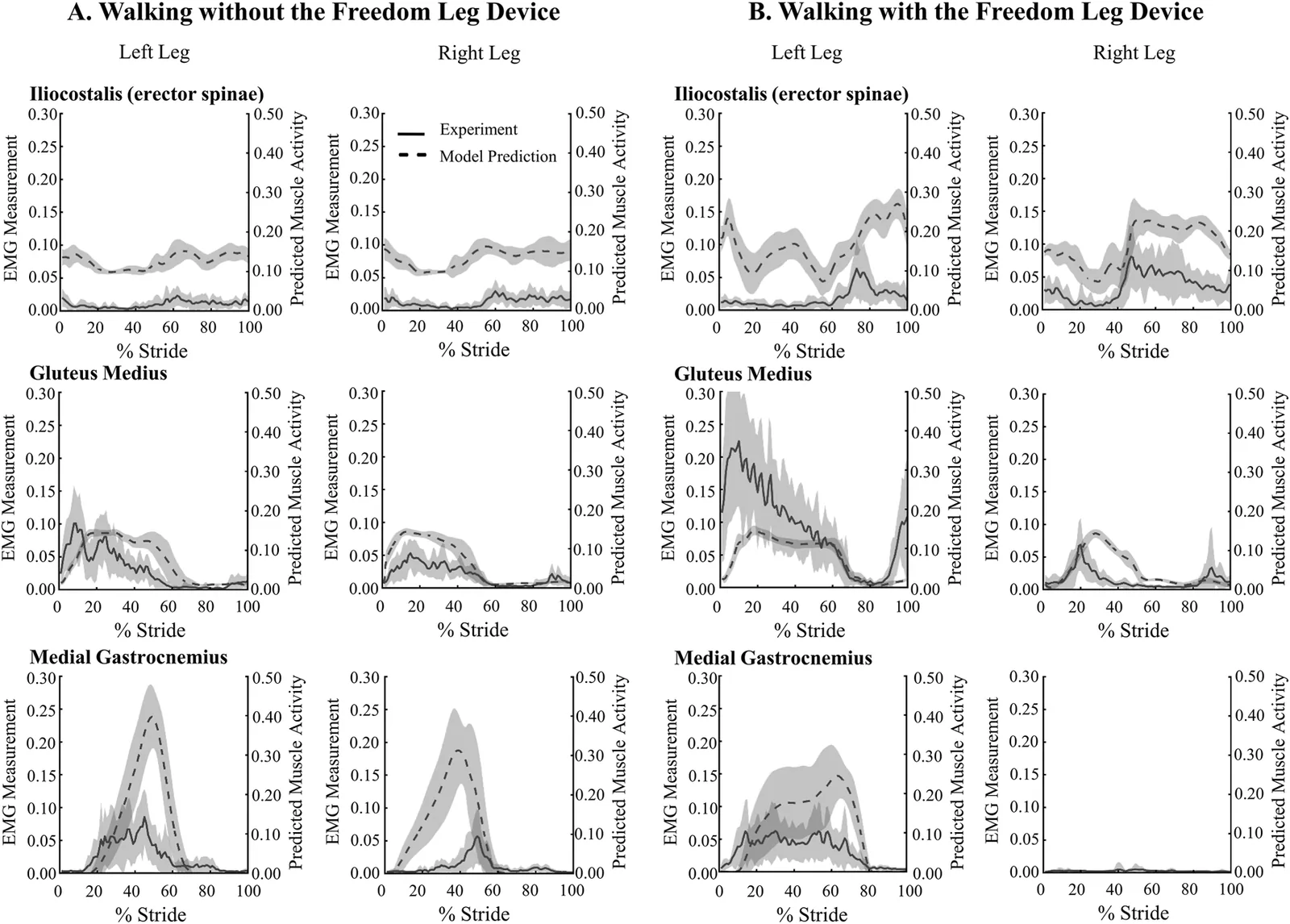
Comparison of muscle activity predicted by the musculoskeletal model (dashed lines) and the electromyography (EMG) data measured during the laboratory experiment (solid lines) for the iliocostalis (erector spinae), gluteus medius, and medial gastrocnemius muscles for one representative participant walking **(A)** without and **(B)** with the Freedom Leg device. For trials with the hands-free crutch, participants wore the device on the right leg. The shaded areas represent the stride-averaged EMG envelopes obtained by low-pass filtering at 20 Hz (mean ± one standard deviation).

### 3.2 Evaluation of the effects of walking with the Freedom Leg device

We evaluated the performance of the Freedom Leg device for its ability to achieve two main objectives: *1*) avoid weight-bearing on the hypothetically injured right leg and *2*) stabilize the tibia of the right leg. In addition, we evaluated the unintended consequences on the contralateral (left) leg resulting from walking with the device on the right leg. To this end, we computed the rank-biserial correlation *r* and *p*-values to assess differences between the two walking conditions (with and without the device) for each of the 36 model-predicted biomechanical and physiological parameters, including spatiotemporal, joint kinematic, and joint kinetic parameters, metabolic power demand, and tibial stress. Based on a literature review, we determined that the most important subset of biomechanical parameters to evaluate the two objectives included knee and ankle JRFs and moments as well as tibial stress. Therefore, we focused our analysis primarily on the subset of the seven parameters listed in Table 2, while considering all 36 parameters in our analysis of unintended consequences.

**TABLE 2:** Biomechanical parameters used to evaluate the performance of the Freedom Leg on the right leg.

Biomechanical parameter	Desired change	References
Objective #1: avoid weight-bearing on the crutch-supported leg		
Peak knee JRF	Decrease	(Hagen et al., 2023; Youdas et al., 2005; Sasaki and Neptune, 2010; D’Lima et al., 2012; De Carvalho et al., 2025; Sukopp et al., 2025)
Peak ankle JRF	Decrease	(Youdas et al., 2005; Ganse et al., 2016; Theodorakos et al., 2022; Xiang et al., 2024; De Carvalho et al., 2025)
Peak tibial stress	Decrease	(Wong et al., 2010; Xu et al., 2016; Hast et al., 2024; Kozin et al., 2026)
Objective #2: stabilize the tibia of the crutch-supported leg		
Peak knee flexion moment	Decrease	(D’Lima et al., 2007; Goldberg and Stanhope, 2013; Andrysek et al., 2014; De Carvalho et al., 2025; Saadat et al., 2026)
Peak knee extension moment	Decrease	(D’Lima et al., 2007; Goldberg and Stanhope, 2013; Andrysek et al., 2014; De Carvalho et al., 2025; Saadat et al., 2026)
Peak ankle plantarflexion moment	Decrease	(Neptune et al., 2001; Goldberg and Stanhope, 2013; Kobayashi et al., 2015; Gao et al., 2024; Saadat et al., 2026)
Peak ankle dorsiflexion moment	Decrease	(Neptune et al., 2001; Goldberg and Stanhope, 2013; Kobayashi et al., 2015; Gao et al., 2024; Saadat et al., 2026)
Peak tibial stress	Decrease	(Wong et al., 2010; Xu et al., 2016; Hast et al., 2024; Kozin et al., 2026)

JRF: joint reaction force.

### 3.3 Assessment of weight-bearing in the crutch-supported leg

To assess whether wearing the Freedom Leg resulted in weight offloading in the lower right leg, we analyzed the GRFs transmitted through the crutch to the lower-extremity joints by comparing the JRFs at the ankle and knee of the right leg when participants walked with and without the device. Compared with the unassisted condition, walking with the Freedom Leg resulted in a significant (*p* = 0.0031) and consistent reduction (average of 98%) in the peak ankle JRF of the right leg of all participants, with a large effect size (ES = −1.00; Table 3; Supplementary Table S1). When participants walked with the device, the peak knee JRF also decreased significantly and consistently, with an average reduction of 47% and a large effect size (ES = −1.00; *p* = 0.0031). The reductions in the JRFs in the knee and ankle, coupled with a significant and large reduction in the peak tibial stress (ES = −1.00; *p* = 0.0031; Table 3; Supplementary Table S1), suggested that minimal load was transferred to the lower right leg, reducing its weight-bearing. Walking with the Freedom Leg reduced the group mean value of each of these parameters by more than two SEM of their unassisted walking values, thereby satisfying the predefined criterion for avoiding weight-bearing. In addition, the peak hip JRF at the right leg also decreased with a large effect size while walking with the Freedom Leg, although it did not reach statistical significance (ES = −0.53; *p* = 0.1860). Overall, the reduced JRFs in the proximal (knee) and distal (ankle) joints of the lower right leg can be attributed to the device’s ability to reduce weight-bearing on the crutch-supported leg, as further evidenced by the negligible activity of the medial gastrocnemius muscle in that leg (Figure 3).

**TABLE 3:** Changes in joint kinetic parameters and tibial stress when walking with the Freedom Leg.

Biomechanical parameter	Left leg			Right leg	
	Effect size (95% CI)		*p*-value	Effect size (95% CI)	*p*-value
Peak ground reaction force (BW)	**1.00 (1.00, 1.00)**		**0.0043**	−0.42 (−1.00, 0.27)	0.3004
Peak joint moment (N·m/kg)					
Hip					
Flexion	**0.89 (0.53, 1.00)**		**0.0149**	1.00 (1.00, 1.00)	**0.0031**
Extension	**1.00 (1.00, 1.00)**		**0.0043**	0.93 (0.67, 1.00)	**0.0081**
Abduction	−0.27 (−1.00, 0.42)		0.5220	−1.00 (−1.00, −1.00)	**0.0031**
Adduction	**1.00 (1.00, 1.00)**		**0.0043**	0.35 (0.42, 0.89)	0.3971
External rotation	**1.00 (1.00, 1.00)**		**0.0043**	0.05 (−0.60, 0.75)	0.9219
Internal rotation	**1.00 (1.00, 1.00)**		**0.0043**	0.78 (0.31, 1.00)	**0.0339**
Knee					
Flexion	−0.49 (−0.96, 0.20)		0.2115	−0.82 (−1.00, −0.27)	**0.0251**
Extension	**1.00 (1.00, 1.00)**		**0.0043**	1.00 (1.00, 1.00)	**0.0031**
Ankle					
Plantarflexion	−0.53 (−1.00, 0.09)		0.1868	−1.00 (−1.00, −1.00)	**0.0031**
Dorsiflexion	**1.00 (1.00, 1.00)**		**0.0043**	−1.00 (−1.00, −1.00)	**0.0031**
Peak joint reaction force (BW)					
Hip	**1.00 (1.00, 1.00)**		**0.0043**	−0.53 (−0.96, 0.16)	0.1860
Knee	0.20 (−0.49, 0.89)		0.6434	−1.00 (−1.00, −1.00)	**0.0031**
Ankle	**1.00 (1.00, 1.00)**		**0.0043**	−1.00 (−1.00, −1.00)	**0.0031**
Tibial stress (MPa)	--	--	--	−1.00 (−1.00, −1.00)	**0.0031**

We evaluated the impact of walking with and without the Freedom Leg by calculating the effect size using the rank-biserial correlation *r* and the *p*-value using the Wilcoxon signed-rank test based on averages of the 10 participants. The values in parentheses show the 95% confidence interval (CI) of the effect size. Negative effect size values indicate that the biomechanical parameter decreased when walking with the Freedom Leg compared to walking unassisted. Use of bold font for *p*-values indicates that the change in the parameter was statistically significant (*p* < 0.05 after applying the Benjamini–Hochberg correction for multiple comparisons). Use of bold font for effect sizes (left leg) indicates that the change in the parameter met the criteria for unintended consequences, i.e., (0.80 ≤ |*r*| ≤ 1.00) and *p* < 0.05. BW: body weight.

### 3.4 Assessment of stability of the tibia in the crutch-supported leg

We assessed the stability of the tibia in the crutch-supported leg by comparing the moments in the proximal (knee) and distal (ankle) joints of the right leg as well as the peak von Mises stress within the tibia when participants walked with and without the Freedom Leg. Compared with the unassisted condition, walking with the device resulted in a statistically significant (*p* = 0.0251) average reduction of 30% in the peak knee flexion moment with a large effect size (ES = −0.82) and a 57% increase in the peak knee extension moment of the right leg (ES = 1.00; *p* = 0.0031) (Table 3; Supplementary Table S1). When participants walked with the device, the peak ankle plantarflexion and dorsiflexion moments decreased significantly to around zero (ES = −1.00), suggesting that the motion of the ankle was drastically reduced in the crutch-supported leg. Except for the peak knee extension moment, all other moments in both the proximal and distal joints of the lower right leg decreased, suggesting a reduced mechanical demand and external loading of the lower leg and possibly reflecting constrained motion of the lower limb during device use. In contrast, the increased peak knee extension moment likely reflected an altered movement strategy (i.e., a flexed knee posture) resulting from the use of the Freedom Leg. In addition, the FE analysis revealed that walking with the device resulted in a significant (*p* = 0.0031) and consistent reduction (average of 59%) in the peak von Mises stress of the right tibia, with a large effect size (ES = −1.00). Walking with the Freedom Leg reduced the group mean value of the peak tibial stress and moments in the ankle and knee by more than two SEM of their unassisted walking values, thereby satisfying the criterion for lower-leg stabilization. The only exception was the increase in the knee extension moment which was likely caused by an altered walking strategy. Taken together, these results suggested that the Freedom Leg can effectively offload and stabilize the shank of the crutch-supported leg.

### 3.5 Assessment of unintended consequences in the left leg

To identify potential unintended consequences on the contralateral (left) leg when participants walked with the Freedom Leg on the right leg, we computed the effect size and assessed the significance of the changes in all parameters, including spatiotemporal, joint kinematic, joint kinetic, and metabolic power demand, between the two walking conditions (Tables 3–5; Supplementary Tables S1-S3). We hypothesized that the contralateral leg would play a substantial role in the compensatory mechanism resulting from offloading and stabilization of the leg wearing the device, leading to potential unintended consequences in the left leg. Here, we defined biomechanical parameters revealing unintended consequences in the left leg as those that yielded meaningful changes between the two conditions by meeting the following criteria: statistically significant differences with *p*-value <0.05 after correction for multiple comparisons and rank-biserial correlation *r* in the range of 0.80 ≤ |*r*| ≤ 1.00.

**TABLE 4:** Changes in spatiotemporal and joint kinematic parameters when walking with the Freedom Leg.

Biomechanical parameter	Left leg		Right leg	
	Effect size (95% CI)	*p*-value	Effect size (95% CI)	*p*-value
Step time (s)	0.49 (−0.20, 1.00)	0.2115	−1.00 (−1.00, −1.00)	**0.0031**
Normalized step length	0.53 (−0.13, 1.00)	0.1868	−1.00 (−1.00, −1.00)	**0.0031**
Peak joint angle and ROM (degree)				
Hip				
Flexion	**1.00 (1.00, 1.00)**	**0.0043**	1.00 (1.00, 1.00)	**0.0031**
Flexion ROM	**0.89 (0.45, 1.00)**	**0.0149**	−0.71 (−1.00, −0.13)	0.0586
Abduction	**1.00 (1.00, 1.00)**	**0.0043**	−1.00 (−1.00, −1.00)	**0.0031**
Abduction ROM	**−1.00 (−1.00, −1.00)**	**0.0043**	−1.00 (−1.00, −1.00)	**0.0031**
External rotation	**1.00 (1.00, 1.00)**	**0.0043**	−1.00 (−1.00, −1.00)	**0.0031**
External rotation ROM	**0.96 (0.78, 1.00)**	**0.0065**	−0.13 (−0.75, 0.64)	0.7915
Knee				
Flexion	0.53 (−0.20, 0.96)	0.1868	0.42 (−0.27, 1.00)	0.3004
Flexion ROM	−0.64 (−1.00, 0.02)	0.1176	−1.00 (−1.00, −1.00)	**0.0031**
Ankle				
Plantarflexion	**0.96 (0.78, 1.00)**	**0.0065**	−0.96 (−1.00, −0.78)	**0.0059**
Plantarflexion ROM	**0.96 (0.78, 1.00)**	**0.0065**	−1.00 (−1.00, −1.00)	**0.0031**
Trunk				
Flexion	**0.96 (0.78, 1.00)**	**0.0065**	0.93 (0.64, 1.00)	**0.0081**
Flexion ROM	**1.00 (1.00, 1.00)**	**0.0043**	1.00 (1.00, 1.00)	**0.0031**

We evaluated the impact of walking with and without the Freedom Leg by calculating the effect size using the rank-biserial correlation *r* and the *p*-value using the Wilcoxon signed-rank test based on averages of the 10 participants. The values in parentheses show the 95% confidence interval (CI) of the effect size. Negative effect size values indicate that the biomechanical parameter decreased after walking with the Freedom Leg compared to walking without it. Use of bold font for *p*-values indicates that the change in the parameter was statistically significant (*p* < 0.05 after applying the Benjamini–Hochberg correction for multiple comparisons). Use of bold font for effect sizes (left leg) indicates that the change in the parameter met the criteria for unintended consequences, i.e., (0.80 ≤ |*r*| ≤ 1.00) and *p* < 0.05. ROM: range of motion.

**TABLE 5:** Changes in metabolic power demand of representative muscles when walking with the Freedom Leg.

Biomechanical parameter	Left Leg		Right Leg	
	Effect size (95% CI)	*p*-value	Effect size (95% CI)	*p*-value
Mean metabolic demand (W/kg)				
Hip extensors				
Gluteus maximus	**1.00 (1.00, 1.00)**	**0.0043**	0.89 (0.56, 1.00)	**0.0130**
Biceps femoris	0.60 (−0.02, 0.96)	0.1367	−1.00 (−1.00, −1.00)	**0.0031**
Hip flexors				
Psoas major	**1.00 (1.00, 1.00)**	**0.0043**	1.00 (1.00, 1.00)	**0.0031**
Rectus femoris	**0.96 (0.78, 1.00)**	**0.0065**	1.00 (1.00, 1.00)	**0.0031**
Hip abductors				
Gluteus medius	0.71 (0.09, 1.00)	0.0712	−1.00 (−1.00, −1.00)	**0.0031**
Hip adductors				
Adductor magnus	0.60 (−0.05, 0.96)	0.1367	1.00 (1.00, 1.00)	**0.0031**
Knee flexors				
Gastrocnemius	−0.13 (−0.78, 0.56)	0.7695	−1.00 (−1.00, −1.00)	**0.0031**

We evaluated the impact of walking with and without the Freedom Leg by calculating the effect size using the rank-biserial correlation *r* and the *p*-value using the Wilcoxon signed-rank test based on averages of the 10 participants. The values in parentheses show the 95% confidence interval (CI) of the effect size. Negative effect size values indicate that the biomechanical parameter decreased after walking with the Freedom Leg compared to walking without it. Use of bold font for *p*-values indicates that the change in the parameter was statistically significant (*p* < 0.05 after applying the Benjamini–Hochberg correction for multiple comparisons). Use of bold font for effect sizes (left leg) indicates that the change in the parameter met the criteria for unintended consequences, i.e., (0.80 ≤ |*r*| ≤ 1.00) and *p* < 0.05.

#### 3.5.1 Spatiotemporal parameters

Walking with the Freedom Leg resulted in longer and slower steps in the left leg compared to the unassisted condition (Supplementary Table S2). The differences did not reach statistical significance but resulted in large effect sizes (Table 4). While we observed these changes in some participants, they were not consistent across all participants, indicating that such gait modulation was not the primary source of compensation. In contrast, we observed significantly shorter and faster steps in the right leg (*p* = 0.0031) with large effect sizes (ES = −1.00), which reflected an expected strategy of the right leg to unload the weight by minimizing the stance duration and forward progression on the affected leg. Therefore, walking with the device resulted in asymmetric step length and step duration between the two legs, with a significant reduction in both parameters in the right leg but no significant changes in the left leg. These results suggest a lack of meaningful unintended consequences in the spatiotemporal parameters of the contralateral leg.

#### 3.5.2 Joint kinematics

Compared to the unassisted condition, walking with the Freedom Leg resulted in significant changes and large effect sizes in the peak joint angles and ROM of the left leg across all three planes of hip motion (Table 4). For example, the peak hip flexion and external rotation angles, as well as their corresponding ROMs, increased significantly (Table 4; Supplementary Table S2). The peak hip abduction angle also increased significantly (ES = 1.00; *p* = 0.0043), but the hip abduction-adduction ROM decreased significantly (ES = −1.00; *p* = 0.0043). In addition, the peak ankle plantarflexion angle and ROM (ES = 0.96; *p* = 0.0065) in the left leg as well as the peak trunk flexion angle (ES = 0.96; *p* = 0.0065) and ROM (ES = 1.00; *p* = 0.0043) increased significantly with large effect sizes when participants walked with the Freedom Leg. These parameters met the criteria for unintended consequences in the contralateral leg, as mentioned above. In contrast, the peak knee flexion angle and ROM did not change significantly but had large effect sizes. These findings indicate that the contralateral hip and ankle as well as the trunk mechanics likely compensated for the altered gait when participants walked with the device.

#### 3.5.3 Joint kinetics

When participants walked with the device, the peak GRF of the left leg increased significantly (*p* = 0.0043) with a large effect size (ES = 1.00; Table 3). Accordingly, the peak JRFs in all three lower-extremity joints (hip, knee, and ankle) also increased. The peak hip and ankle JRFs increased significantly (*p* = 0.0043) and consistently across all participants by ∼19% with large effect sizes (ES = 1.00), whereas the peak knee JRF did not reach statistical significance and had a small effect size (ES = 0.20). The increased hip JRF in the left leg was consistent with the contralateral hip discomfort reported by participants and with the elevated muscle activity in the corresponding gluteus medius discussed above (Figure 3B).

Consistent with the changes observed in the peak hip joint angles in Table 4, the peak hip joint moments across all degrees of freedom increased significantly (*p* < 0.0149) with large effect sizes (ES > 0.89), except for the peak hip adduction moment (Table 3; Supplementary Table S1). The peak knee flexion moment did not change significantly, but the peak knee extension moment increased significantly (*p* = 0.0043) and consistently across all participants, with a large effect size (ES = 1.00). Similarly, the peak ankle plantarflexion moment did not change significantly, but the peak ankle dorsiflexion moment increased significantly (*p* = 0.0043) and consistently in all 10 participants, with a large effect size (ES = 1.00). We were particularly interested in meaningful changes in joint moments and JRFs because these alterations may reflect adaptations in muscle recruitment when participants walked with the Freedom Leg and may provide insights into changes in mechanical and energetic demand throughout the gait cycle.

#### 3.5.4 Metabolic power demand

Without exception, the predicted metabolic power demand for the left leg increased across all major hip muscle groups when participants walked with the Freedom Leg compared to the unassisted condition (Table 5; Supplementary Table S3). All these increases showed large effect sizes based on rank-biserial correlation (ES > 0.43). However, these increases only reached statistical significance in the left gluteus medius (*p* = 0.0043), psoas major (*p* = 0.0043), and rectus femoris (*p* = 0.0065). Metabolic power demand also increased in several hip muscles in the right leg, including the gluteus maximus, psoas major, rectus femoris, and adductor magnus, however, these increases were accompanied by a contrasting decrease in other muscle groups, including the biceps femoris and gluteus medius. Overall, the elevated metabolic power demand in both legs indicated greater effort during device-assisted walking and was consistent with the observed alterations in joint kinetics. The metabolic power demand in the left knee flexor (gastrocnemius) muscle did not change significantly (ES = −0.13; *p* = 0.7695), yet it decreased to negligible levels in the right knee flexor muscle (ES = −1.00; *p* = 0.0031), reflecting offloading of the right lower leg when participants walked with the device.

## 4 Discussion

In this exploratory study, we aimed to assess the performance of the Freedom Leg hands-free crutch for its ability to aid in the mobility of an individual with a hypothetical tibial injury by avoiding weight-bearing on the crutch-supported leg and stabilizing the injury. To this end, we collected experimental data, including anthropometric, body-motion, GRF, spatiotemporal, and EMG measurements from 10 healthy men as they walked on an instrumented treadmill with and without the device. Using these experimental data, we developed and verified MSK models to predict joint mechanics and muscle activity in both legs and the lower back and developed 3-D FE models of the crutch-supported right tibia to compute peak stress for each walking condition. To evaluate the performance of the Freedom Leg, we quantified the forces transmitted to the lower-extremity joints and the extent of unintended consequences by comparing the model-predicted biomechanical parameters obtained under the two conditions, for both the crutch-supported leg and the contralateral leg. We hypothesized that the use of the Freedom Leg would reduce JRFs and moments at the knee and ankle joints as well as decrease tibial stress for the crutch-supported right leg. We also hypothesized that the device would cause unintended consequences by increasing JRFs, moments, and muscle activity for the contralateral leg. In agreement with these hypotheses, the Freedom Leg effectively aided in the mobility of individuals wearing the device by reducing the peak JRFs at the knee by 47% and at the ankle by 98% of the right leg, while reducing the peak tibial stress by 59%. In contrast, walking with the device introduced several unintended consequences, including a 19% increase in the peak hip JRF in the contralateral leg.

### 4.1 The Freedom Leg reduced weight-bearing and stabilized the crutch-supported lower leg

We observed significant reductions in knee and ankle JRFs for the crutch-supported right leg, which suggest that, as previously reported for standard crutches (Clark et al., 2004; Damm et al., 2013; Saadat et al., 2026), the use of the device shifts the impact with the ground away from the hypothetically injured lower extremity to the crutch and reduces weight-bearing. We also observed significant reductions in the peak ankle moments, which approached negligible values, and the peak knee flexion moment, while noticing significant increases in the knee extension moment (Table 3; Supplementary Table S1). The decrease in knee flexion and ankle moments suggest a reduction in the internal muscle forces in the corresponding muscle groups around the hypothetically injured leg. The reduced muscle forces around the injured limb may reflect decreased muscular demand to counteract external loads, suggesting a reduction in dynamic internal joint loading during gait. The increase in knee extension moment of the crutch-supported leg reflects greater muscular demand and reliance on the knee extensors to stabilize the leg during the stance phase, enhancing the structural stability when the knee flexion ROM is significantly reduced (Table 4; Supplementary Table S1). This increase can be attributed to a passive compensatory strategy to maintain knee stability in the absence of ankle support. In addition, as the load is distributed to the crutch instead of the ankle, the right ankle’s mechanical demand decreases and the crutch effectively shields the ankle from forces that would normally be experienced by the leg. Our FE analysis further supports this interpretation, with a significant reduction in peak von Mises stress of the right tibia, which indicates a substantial offloading of the crutch-supported lower leg (Table 3; Supplementary Table S1). This enhances mechanical stability in the hypothetically injured tibia and, as a consequence, establishes a biomechanical environment to shield the injured zone and promote steady healing (Hast et al., 2024).

### 4.2 Comparison with previous hands-free crutch studies on the crutch-supported leg

The novelty of our work lies in the implementation of an integrated MSK/FE framework to investigate the performance of the Freedom Leg hands-free crutch. Previous biomechanical studies of hands-free crutches have been limited to the iWalk device (Rambani et al., 2007; Dewar and Martin, 2020; Dewar et al., 2021; Orishimo et al., 2021; Kim et al., 2023; Canter et al., 2024; Hegeman and Huh, 2024; Saffuri et al., 2024), and none performed MSK analyses like the one reported herein. As such, these investigations inferred the responses of biomechanical parameters either through direct measurements or through simulations based on simple rigid-body models (linked-segment models), which do not account for muscle responses and lack a realistic representation of the human anatomy, preventing a comprehensive examination of kinetic parameters in the lower-leg joints. In contrast, we developed full-body, personalized MSK models to assess lower-extremity joint kinetics and kinematics and muscle metabolic demand when participants walked with the Freedom Leg. In addition, none of the previous studies developed FE models to investigate the stress distribution in the theoretically injured limb supported by the iWalk or any other hands-free device. In contrast, we developed personalized FE models to predict the peak stress in the crutch-supported right tibia, which allowed us to quantify the offloading and leg-stabilization capabilities of the Freedom Leg. Importantly, the Freedom Leg and the iWalk devices are based on distinct designs with different weight-offloading patterns. Taken together, these differences in parameter estimation and hands-free crutch design prevent a one-to-one comparison of gait patterns. Nevertheless, to provide context to our findings, in what follows we discuss our results in light of reported observations for the iWalk device.

Orishimo et al., whose analysis was limited to the crutch-supported leg based on a linked-segment model, found that when healthy participants wore the iWalk hands-free crutch, they experienced a lower hip JRF than when walking unassisted (Orishimo et al., 2021). We observed a similar pattern and large effect size with the Freedom Leg device, although the decrease did not reach statistical significance (Table 3; Supplementary Table S1). They also reported an 18% increase in the reaction force in the lower back when walking with the iWalk device (Orishimo et al., 2021). We did not quantify the reaction force in the lower back, however, we qualitatively observed an elevated muscle activity in the erector spinae muscles when using the Freedom Leg device, which corroborates their finding (Figure 3; Supplementary Figure S10). This observation is consistent with an elevated lower-back reaction force (Orishimo et al., 2021) and reports of lower-back pain while walking with the iWalk, as observed in a randomized controlled trial by Rambani et al. (Rambani et al., 2007). Kinematically, Orishimo et al. found that, compared with the unassisted condition, walking with the iWalk decreased the hip ROM and increased the trunk ROM in both the sagittal and frontal planes of the affected limb (Orishimo et al., 2021). We observed similar trends in the hip and trunk with the Freedom Leg, however, the decrease in the hip flexion ROM did not reach statistical significance (*p* = 0.0586). In addition, Orishimo et al. reported a 12% decrease in the vertical GRF of the affected leg when walking with the device (Orishimo et al., 2021), while we observed nearly identical GRF amplitudes with and without the device. This suggests that the Freedom Leg possibly facilitates a more natural contact with the ground in the affected side of the body while effectively offloading the injured region.

### 4.3 Unintended consequences and comparison with previous hands-free crutch studies on the contralateral leg

We found that walking with the Freedom Leg resulted in unintended consequences on the contralateral leg, especially in the biomechanics of the hip and ankle joints. Compared with the unassisted condition, walking with the Freedom Leg significantly increased the peak hip JRF by 19% and the hip flexion ROM by 11% as well as the hip flexion and extension moments of the left leg by 50% and 89%, respectively, with large effect sizes (ES ≥ 0.89) (Table 3; Supplementary Table S1). These increases led to enhanced muscle activity and joint loading on the contralateral hip reflect a compensatory mechanism caused by the reduced functionality of the lower leg in the crutch-supported limb. Similarly, we also observed large effect sizes and significant increases in the peak ankle JRF and plantarflexion ROM (Table 4; Supplementary Table S2). In contrast, Kim et al., who only analyzed the effect of walking with the iWalk on the contralateral leg, reported that, compared with walking unassisted, walking with the device resulted in similar knee and ankle flexion ROM (Kim et al., 2023). In addition, instead of the observed significant increase in hip flexion ROM in our study, they observed a significant decrease. These discrepancies are likely attributed to the different methods used for parameter estimation (stride-specific value vs. phase-specific value within the stride), gait speed between conditions, and device design, leading to different weight-offloading patterns. We also observed an increase in the trunk flexion ROM, possibly because of a compensatory mechanism as evidenced by an increase in the activation of the erector spinae muscle on the contralateral leg (Figure 3).

In the contralateral leg, the metabolic power demand of representative hip muscles increased with large effect sizes, however, the increases did not reach statistical significance for all muscles. The metabolic power demand also increased in several muscles of the crutch-supported leg. These increases are consistent with the participants’ consistently elevated RPE (nine out of the 10 participants) when walking with the Freedom Leg compared with unassisted walking. These findings are also consistent with previous reports of a higher energy expenditure and RPE when walking with the iWalk hands-free crutch compared with unassisted walking (Canter et al., 2024). Overall, the Freedom Leg did not reduce the loading of the knee and ankle joints of the crutch-supported leg by overloading its hip. Rather, the Freedom Leg compensated for the support provided by the contralateral limb by overloading this limb, especially the hip. Indeed, the participants’ subjective comments reporting contralateral hip discomfort and increased cognitive effort when wearing the hands-free crutch are consistent with our MSK and FE modeling results.

### 4.4 Use of the Freedom Leg resulted in an asymmetric walking pattern

Walking with the Freedom Leg resulted in asymmetric step length and step duration between the two legs, with a statistically significant reduction in both parameters in the right leg and non-significant increases in the left leg, to maintain gait speed. Asymmetrical, altered walking patterns are well-established protective mechanisms to minimize weight-bearing, discomfort, and pain on injured lower limbs (Federer, 2025). The reduction of the spatiotemporal parameters in the crutch-supported leg correlates with a reduced stance phase (Nonnekes et al., 2025) and is associated with an antalgic or cautious gait. We observed a similar altered gait pattern in our study (Supplementary Figure S11). In particular, walking with the hands-free crutch led to a reduced stance duration and a prolonged swing duration in the affected limb. In agreement with our findings, Kim et al. observed a significant increase in stride time in the contralateral leg, however, their observation of a significant decrease in stride length contradicts our findings (Kim et al., 2023). This discrepancy is likely attributed to the fact that we standardized the gait speed across the two conditions, while Kim et al. allowed participants to decrease their preferred speed when walking with the iWalk device. Nevertheless, similar to our findings (Supplementary Figure S11), Kim et al. also observed a prolonged stance duration and a reduced swing duration in the contralateral leg while walking with the hands-free crutch.

### 4.5 Advantages of the Freedom Leg and other potential applications

A few commercially available hands-free devices allow for ambulation, while avoiding weight-bearing on the foot or lower leg and keeping the upper extremities free from managing the device (Rasouli and Reed, 2020; Hegeman and Huh, 2024). For example, ankle-foot orthosis (AFO) devices are designed to transfer GRFs to the shank via braces tightened around it, while completely offloading the affected foot (Saffuri et al., 2024). Although AFO devices offer a more symmetric and natural walking pattern with a lower metabolic cost than the iWalk (Saffuri et al., 2024), similar to the iWalk, they are not suitable for lower-leg injuries proximal to the tibia because these types of devices transmit load through the shank. In contrast, similar to the XLeg (Robinson Exotech Corporation, 2026), the Freedom Leg is a hands-free crutch suitable for injuries both proximal and distal to the tibia, including the knee, shank, ankle, and foot, because it offloads all of these lower-limb regions of the human body. As such, in addition to extremity wounds and fractures, the Freedom Leg can also be beneficial for stress fracture of the tibia, one of the most prevalent MSK injuries in military personnel and athletes alike (Wood et al., 2014; Kahanov et al., 2015), because tibial stress fracture requires a non-weight-bearing rehabilitation period of about 6–12 weeks, depending on the grade and location of the injury (Kahanov et al., 2015).

### 4.6 Study limitations

This study has limitations. First, we recruited only healthy participants and assumed that having such participants walk with the Freedom Leg would result in the same gait biomechanics as if they had an injured lower leg. Healthy individuals lack altered motor-control strategies, neuromuscular adaptations, and pain-induced antalgic gait patterns experienced by injured cohorts. However, recruiting injured participants for biomechanical testing of an aid-assisted device on an instrumented treadmill introduces substantial ethical concerns and safety risks. Taken together, the recruitment of healthy participants and the small sample size may limit the generalizability of our findings to individuals with lower-leg injuries. Nevertheless, we attempted to minimize this potential limitation by setting and fixing the walking speed for each participant to those obtained when participants walked at their preferred speed with the device. Second, each participant completed the walking trials in the same order, first with the device and then without it, which may have introduced potential fatigue and carryover effects. However, we expect these effects to be negligible because each walking trial was short (40 s long, consisting of two 20-s segments separated by approximately 2 min) and participants rested for at least 5 min between trials, with additional rest provided as needed before the second trial. Third, we did not explicitly model the straps of the thigh and calf of the Freedom Leg. Instead, we implicitly modeled them by distributing their weight on the side rails of the hands-free crutch device. This approximation should not affect the MSK model predictions because the straps weigh much less than the side rails (∼0.2 kg vs. ∼2.0 kg) of the device.

Fourth, the assessment of the personalized MSK models was limited to a qualitative comparison between experimentally measured EMG recordings and model-predicted muscle activities, as these parameters represent different quantities and do not permit direct quantitative comparison. However, the model-predicted activity of major muscle groups showed a qualitative agreement with the measured EMG recordings, indicating that the personalized MSK models reasonably reproduced the expected timing and relative magnitude of muscle activation when walking with and without the Freedom Leg. Fifth, regarding the coupling of the hands-free crutch device with the MSK model, we assumed that there was no relative translational motion between the upper leg and the side rails and that the relative translational motion between the lower leg and the side rails was limited to the vertical direction. Motion-capture data and analysis of video recordings supported this assumption because we observed only minimal relative motion. Finally, to develop the FE model of the tibia, we assumed a uniform cortical bone thickness and the same material properties for all participants, because we lacked computed tomography scans of their tibia. Although accounting for participant-specific cortical-bone distribution and material properties is important for quantifying stress-fracture risk at the individual level (Rubio et al., 2023; Tong et al., 2023), this approximation is reasonable because here we only used FE models to assess relative changes in stress between crutch conditions within a participant, which is not dependent on bone properties.

### 4.7 Future directions

Wearable exoskeleton devices have emerged as novel tools to restore body mobility, augment physical strength, and help prevent injury (Johnson et al., 2023; McGrath et al., 2024). In addition, exoskeletons offer the potential to restore mobility debilitated by lower-extremity long-bone fracture or lower-leg wound (Johnson et al., 2023; McGrath et al., 2024). However, conventional exoskeleton devices are not suitable for the latter application because they lack the ability to avoid weight-bearing on the injured leg. To overcome this limitation and potentially reduce the unintended consequences of hands-free crutches, exoskeleton designs can draw inspiration from mobility-aid devices, which provide fracture stabilization and leg support, while relieving weight-bearing from the injured leg by redirecting loads to nearby upper-leg tissues (Franklin et al., 2019; Johnson et al., 2023). For example, by combining the augmentation capabilities of hip-assisted exoskeletons, which have been shown to reduce metabolic costs and improve gait balance (Bryan et al., 2021; Zhou et al., 2021; Yang et al., 2022), with the beneficial features of hands-free crutches, a novel exoskeleton device could provide superior mobility functionality for Service members with lower-leg wounds or fracture.

## 5 Conclusion

In this exploratory interdisciplinary study, we quantified the performance of the Freedom Leg hands-free crutch to aid in the mobility of healthy individuals with a hypothetical lower-leg injury proximal to the tibia. We found that the Freedom Leg effectively reduced the JRFs and moments in the ankle and knee as well as the stress in the tibia of the affected leg, confirming its beneficial use in stabilizing the injury and avoiding weight-bearing on the crutch-supported leg. However, we also found that offloading the injured leg led to compensatory mechanisms and changes in gait patterns on the contralateral side of the body with unintended consequences in the contralateral hip. In summary, this study provides insights into the biomechanical effects of walking with the Freedom Leg in healthy, young individuals and may inform the development of future hands-free crutch devices to improve the mobility of Service members with lower-leg injuries on the battlefield and during clinical rehabilitation in the civilian sector.

## Data Availability

The original contributions presented in the study are included in the article/Supplementary Material, further inquiries can be directed to the corresponding author.
