# Supplementary material for: Characterization of lower-extremity biomechanics when walking with a hands-free crutch: an integrated experimental and computational analysis
Source: Front Bioeng Biotechnol. 2026 Sep 30;14:1941015. doi: 10.3389/fbioe.2026.1941015 (PMC13661938; doi:10.3389/fbioe.2026.1941015)
Supplement: Supplementary file 1 [file DataSheet1.docx]

Supplementary Material

Characterization of lower-extremity biomechanics when walking with a hands-free crutch: an integrated experimental and computational analysis

Md. Arifuzzaman Arif^1,2^, Jaques Reifman^1*^, Ankur Padhye^3^, Stacey Meardon^3^, John Willson^3^,
and Jose E. Rubio^1,2^

^1^Department of Defense Biotechnology High Performance Computing Software Applications Institute, Defense Health Agency Research & Development, Medical Research and Development Command, Fort Detrick, MD, USA

^2^The Henry M. Jackson Foundation for the Advancement of Military Medicine, Inc., Bethesda, MD, USA

^3^Department of Physical Therapy, East Carolina University, Greenville, NC, USA

*Correspondence:

Jaques Reifman, Ph.D.

Department of Defense Biotechnology High Performance Computing Software Applications Institute

Defense Health Agency Research & Development

Medical Research and Development Command

ATTN: FCMR-TT, 504 Scott Street

Fort Detrick, MD 21702-5012

E-mail: jaques.reifman.civ@health.mil

Phone: + 1 301 639 3637

Fax: +1 301 619 1983

# 1 Supplementary Tables

**Supplementary Table S1.** Joint kinetic parameters and tibial stress when walking with and without the Freedom Leg

| **Biomechanical**  **Parameter** | **Left Leg** | | **Right Leg** | |
| --- | --- | --- | --- | --- |
|  | **Without Freedom Leg** | **With Freedom Leg** | **Without Freedom Leg** | **With Freedom Leg** |
| Peak ground reaction force (BW) | 1.03 ± 0.02 | 1.17 ± 0.07 | 1.04 ± 0.02 | 1.03 ± 0.03 |
| Peak joint moment (N·m/kg) |  |  |  |  |
| Hip |  |  |  |  |
| Flexion | 0.16 ± 0.04 | 0.24 ± 0.06 | 0.17 ± 0.05 | 0.32 ± 0.05 |
| Extension | 0.57 ± 0.09 | 1.08 ± 0.29 | 0.58 ± 0.09 | 0.84 ± 0.14 |
| Abduction | 0.80 ± 0.06 | 0.75 ± 0.17 | 0.73 ± 0.05 | 0.48 ± 0.10 |
| Adduction | 0.02 ± 0.03 | 0.16 ± 0.08 | 0.04 ± 0.06 | 0.06 ± 0.04 |
| External rotation | 0.11 ± 0.05 | 0.34 ± 0.10 | 0.12 ± 0.05 | 0.13 ± 0.05 |
| Internal rotation | 0.02 ± 0.01 | 0.04 ± 0.02 | 0.01 ± 0.01 | 0.02 ± 0.02 |
| Knee |  |  |  |  |
| Flexion | 0.40 ± 0.17 | 0.35 ± 0.16 | 0.23 ± 0.09 | 0.16 ± 0.04 |
| Extension | 0.16 ± 0.11 | 0.52 ± 0.23 | 0.30 ± 0.12 | 0.47 ± 0.19 |
| Ankle |  |  |  |  |
| Plantarflexion | 1.01 ± 0.17 | 0.95 ± 0.10 | 1.06 ± 0.17 | 0.00 ± 0.00 |
| Dorsiflexion | 0.09 ± 0.04 | 0.18 ± 0.04 | 0.07 ± 0.04 | 0.01 ± 0.00 |
| Peak joint reaction force (BW) |  |  |  |  |
| Hip | 3.69 ± 0.45 | 4.38 ± 0.42 | 3.29 ± 0.31 | 3.03 ± 0.65 |
| Knee | 3.09 ± 0.69 | 3.20 ± 0.35 | 2.76 ± 0.23 | 1.46 ± 0.34 |
| Ankle | 5.08 ± 0.73 | 5.97 ± 0.60 | 4.98 ± 0.71 | 0.11 ± 0.02 |
| Peak tibial stress (MPa) | -- | -- | 48.64 ± 7.96 | 19.71 ± 1.64 |

The data are presented as mean [±1 standard deviation (SD)]. BW, body weight.

**Supplementary Table S2.** Spatiotemporal and joint kinematic parameters when walking with and without the Freedom Leg

| **Biomechanical**  **Parameter** | **Left Leg** | | **Right Leg** | |
| --- | --- | --- | --- | --- |
|  | **Without Freedom Leg** | **With Freedom Leg** | **Without Freedom Leg** | **With Freedom Leg** |
| Step time (s) | 0.80 ± 0.10 | 0.84 ±  0.11 | 0.77 ± 0.09 | 0.64 ± 0.06 |
| Normalized step length | 0.26 ± 0.03 | 0.27 ±  0.04 | 0.25 ± 0.03 | 0.21 ± 0.04 |
| Peak joint angle and ROM (degree) |  |  |  |  |
| Hip |  |  |  |  |
| Flexion | 24.23 ± 5.41 | 33.40 ±  8.12 | 27.74 ± 4.07 | 49.08 ± 4.85 |
| Flexion ROM | 31.79 ± 2.81 | 35.42 ±  4.43 | 31.87 ± 3.44 | 27.74 ± 5.93 |
| Abduction | 14.43 ± 2.51 | 17.40 ±  2.07 | 17.45 ± 3.96 | 9.23 ± 3.15 |
| Abduction ROM | 19.01 ± 3.66 | 11.52 ±  2.10 | 19.49 ± 4.30 | 9.00 ± 2.35 |
| External rotation | 13.90 ± 5.21 | 19.93 ±  4.91 | 13.91 ± 5.53 | 6.12 ± 4.24 |
| External rotation ROM | 8.57 ± 2.56 | 13.29 ±  3.42 | 9.25 ± 3.22 | 8.49 ± 1.79 |
| Knee |  |  |  |  |
| Flexion | 67.92 ± 4.42 | 71.75 ±  5.33 | 70.79 ± 3.76 | 72.98 ± 4.55 |
| Flexion ROM | 61.15 ± 4.60 | 58.14 ±  4.63 | 55.29 ± 2.51 | 12.34 ± 3.87 |
| Ankle |  |  |  |  |
| Plantarflexion | 10.45 ± 4.04 | 18.45 ± 10.43 | 7.23 ± 4.83 | −8.05 ± 7.38* |
| Plantarflexion ROM | 23.47 ± 3.78 | 34.04 ± 11.48 | 23.35 ± 3.25 | 8.71 ± 4.42 |
| Trunk |  |  |  |  |
| Flexion | 11.59 ± 2.27 | 18.69 ±  7.14 | 11.58 ± 2.44 | 18.16 ± 6.71 |
| Flexion ROM | 4.02 ± 0.72 | 8.57 ±  3.67 | 4.10 ± 0.79 | 8.17 ± 3.34 |

The data are presented as mean [±1 standard deviation (SD)]. ROM: range of motion. *No true plantarflexion occurred in the right leg while walking with the Freedom Leg, as the joint angle remained dorsiflexed (negative) throughout the stride.

**Supplementary Table S3.** Metabolic power in representative muscle groups when walking with and without the Freedom Leg

| **Biomechanical**  **Parameter** | **Left Leg** | | **Right Leg** | |
| --- | --- | --- | --- | --- |
|  | **Without Freedom Leg** | **With Freedom Leg** | **Without Freedom Leg** | **With Freedom Leg** |
| Mean metabolic power (W/kg) |  |  |  |  |
| Hip extensors |  |  |  |  |
| Gluteus maximus | 0.07 ± 0.04 | 0.27 ± 0.16 | 0.12 ± 0.06 | 0.18 ± 0.04 |
| Biceps femoris | 0.33 ± 0.17 | 0.44 ± 0.29 | 0.23 ± 0.09 | 0.10 ± 0.05 |
| Hip flexors |  |  |  |  |
| Psoas major | 0.04 ± 0.02 | 0.08 ± 0.03 | 0.05 ± 0.02 | 0.10 ± 0.03 |
| Rectus femoris | 0.02 ± 0.01 | 0.05 ± 0.02 | 0.03 ± 0.01 | 0.06 ± 0.02 |
| Hip abductors |  |  |  |  |
| Gluteus medius | 0.29 ± 0.04 | 0.34 ± 0.08 | 0.27 ± 0.04 | 0.14 ± 0.03 |
| Hip adductors |  |  |  |  |
| Adductor magnus | 0.06 ± 0.03 | 0.10 ± 0.08 | 0.08 ± 0.03 | 0.18 ± 0.05 |
| Knee flexors |  |  |  |  |
| Gastrocnemius | 0.23 ± 0.09 | 0.21 ± 0.08 | 0.28 ± 0.10 | 0.00 ± 0.00 |

The data are presented as mean [±1 standard deviation (SD)].

# 2 Supplementary Figures

**Supplementary Figure S1.** Comparison of muscle activity predicted by the musculoskeletal model (dashed lines) and the electromyography (EMG) data measured during the laboratory experiment (solid lines) for the iliocostalis (erector spinae), gluteus medius, and medial gastrocnemius muscles for participant #1 walking (A) without and (B) with the Freedom Leg device.

**Supplementary Figure S2.** Comparison of muscle activity predicted by the musculoskeletal model (dashed lines) and the electromyography (EMG) data measured during the laboratory experiment (solid lines) for the iliocostalis (erector spinae), gluteus medius, and medial gastrocnemius muscles for participant #2 walking (A) without and (B) with the Freedom Leg device.

**Supplementary Figure S3.** Comparison of muscle activity predicted by the musculoskeletal model (dashed lines) and the electromyography (EMG) data measured during the laboratory experiment (solid lines) for the iliocostalis (erector spinae), gluteus medius, and medial gastrocnemius muscles for participant #3 walking (A) without and (B) with the Freedom Leg device.

**Supplementary Figure S4.** Comparison of muscle activity predicted by the musculoskeletal model (dashed lines) and the electromyography (EMG) data measured during the laboratory experiment (solid lines) for the iliocostalis (erector spinae), gluteus medius, and medial gastrocnemius muscles for participant #4 walking (A) without and (B) with the Freedom Leg device.

**Supplementary Figure S5.** Comparison of muscle activity predicted by the musculoskeletal model (dashed lines) and the electromyography (EMG) data measured during the laboratory experiment (solid lines) for the iliocostalis (erector spinae), gluteus medius, and medial gastrocnemius muscles for participant #5 walking (A) without and (B) with the Freedom Leg device.

**Supplementary Figure S6.** Comparison of muscle activity predicted by the musculoskeletal model (dashed lines) and the electromyography (EMG) data measured during the laboratory experiment (solid lines) for the iliocostalis (erector spinae), gluteus medius, and medial gastrocnemius muscles for participant #6 walking (A) without and (B) with the Freedom Leg device.

**Supplementary Figure S7.** Comparison of muscle activity predicted by the musculoskeletal model (dashed lines) and the electromyography (EMG) data measured during the laboratory experiment (solid lines) for the iliocostalis (erector spinae), gluteus medius, and medial gastrocnemius muscles for participant #7 walking (A) without and (B) with the Freedom Leg device.

**Supplementary Figure S8.** Comparison of muscle activity predicted by the musculoskeletal model (dashed lines) and the electromyography (EMG) data measured during the laboratory experiment (solid lines) for the iliocostalis (erector spinae), gluteus medius, and medial gastrocnemius muscles for participant #8 walking (A) without and (B) with the Freedom Leg device.

**Supplementary Figure S9.** Comparison of muscle activity predicted by the musculoskeletal model (dashed lines) and the electromyography (EMG) data measured during the laboratory experiment (solid lines) for the iliocostalis (erector spinae), gluteus medius, and medial gastrocnemius muscles for participant #10 walking (A) without and (B) with the Freedom Leg device

**Supplementary Figure S10.** Comparison of the electromyography (EMG) signals when walking unassisted (blue) and with a Freedom Leg (red) for the biceps femoris, medial gastrocnemius, gluteus medius, rectus femoris, and iliocostalis (erector spinae) muscles for one representative subject. We normalized the EMG signals to peak EMG values to facilitate comparison between the conditions. The shaded areas represent the stride-averaged EMG envelopes (mean ± one standard deviation).

**Supplementary Figure S11.** Comparison of the vertical ground reaction force (GRF) when walking unassisted (blue) and with a Freedom Leg (red) for one representative subject. The shaded areas represent the stride-averaged GRF values (mean ± one standard deviation). BW, body weight.
